# Mediation effect of emotional self-regulation in the relationship between physical activity and subjective well-being in Chilean adolescents

**DOI:** 10.1038/s41598-023-39843-7

**Published:** 2023-08-17

**Authors:** Sergio Fuentealba-Urra, Andrés Rubio, Mònica González-Carrasco, Juan Carlos Oyanedel, Cristian Céspedes-Carreno

**Affiliations:** 1 Facultad de Educación y Ciencias Sociales, Universidad Andres Bello, 4030000 Concepción, Chile; 2 Facultad de Economia y Negocios, Universidad Andres Bello, Fernandez Concha 700, Las Condes, Santiago Chile; 3 Facultad de Psicología, Universidad Diego Portales, Santiago, Chile; 4 Research Institute on Quality of Life (IRQV), Universitat de Girona, Girona, Spain

**Keywords:** Psychology, Health care

## Abstract

Adolescents' subjective well-being and physical activity have been found to be correlated in previous studies. However, the underlying mechanisms of this relationship, especially the potential contribution of emotional self-regulation, have received little attention. This study aims to investigate the extent to which emotional self-regulation mediates the association between adolescent physical activity habits and their subjective well-being. The study involved 9585 adolescents who completed a cross-sectional survey. Participants were aged between 10 and 19 years old and attended primary and secondary schools in all 16 regions of Chile. The survey utilized a self-report questionnaire to measure physical activity habits, subjective well-being, and emotional self-regulation. Sociodemographic variables, such as age, gender, and socioeconomic level, were also considered in the analysis. The results showed that physical activity habits, emotional regulation, and subjective well-being were positively correlated. Among these factors, the strongest association was found between subjective well-being and emotional self-regulation. The mediation analysis revealed a partial mediation effect of emotional self-regulation between physical activity habits and subjective well-being. In other words, physical activity habits affect subjective well-being to the extent that these habits affect emotional self-regulation. These findings provide valuable insights into the mechanisms underlying the link between physical activity habits and subjective well-being among adolescents. They also offer useful information for the development of public programs and policies aimed at promoting physical activity habits and subjective well-being in young people.

## Introduction

Adolescence is a period of significant transformation characterized by substantial changes in social roles and biological growth that occur between childhood and adulthood^1^. According to the United Nations Children's Fund (UNICEF), this developmental stage lasts from 10 to 20 years old and is further subdivided into early adolescence (10–12 years old), middle adolescence (13–15 years old), and late adolescence (16–20 years old)^2^. Adolescence is widely considered to be a critical phase during which significant physical, mental, and emotional changes take place, laying the groundwork for adult health^3–5^. Adolescents typically have greater control over their activities during this period, and their level of well-being is closely linked to their activities^6,7^.

Subjective well-being (SWB) is the most commonly used approach to assess well-being. This concept is rooted in a positive perspective and has facilitated research into people's behavior and quality of life, as well as the factors that influence these aspects. SWB refers to the way in which individuals assess their lives, both generally and in relation to specific domains^8^. From a hedonic perspective, SWB comprises life satisfaction, positive affect, and negative affect^9^. Over time, a four-part conception has emerged regarding the level of satisfaction that people express about various aspects of their lives, including friends, family, and free time^10–12^. According to Cummins and Cahill^13^, SWB is based on cognitive and affective processes that are unique to each individual and arise from their interpretation of positive or negative emotional experiences^14–16^.

Adolescents' subjective well-being (SWB) is influenced by numerous factors, including healthy lifestyle habits^17^. The literature highlights a close and consistent relationship between SWB and physical activity habits (PAH) in adolescents^18,19^. Bouchard et al.^20^ define physical activity as any movement that requires a considerable amount of energy expenditure and manifests in adolescence through play, sport, exercise, outdoor activities, and physical education programs^21^. Hagger^22^ defines habits as specific behavioral responses that occur simultaneously with environmental cues or contextual characteristics.

Research has shown that engaging in regular physical activity across a variety of circumstances is linked to higher levels of subjective well-being, which in turn are related to a higher quality of life^23,24^. The literature suggests the notion of affective regulation as one of the variables that may mediate between SWB and physical activity^25–27^. Physical activity improves mood and reduces irritation, anxiety, and stress, while also modifying and regulating neurobiological and psychological processes that generally have a positive impact on adolescents' well-being^28^.

The transition from childhood to adolescence is marked by an increase in the quantity and complexity of interactions across a range of contexts. As a result, and in line with the concept of affective regulation, young people need to expand their repertoire of self-regulatory abilities to assist their development and the formation of new and improved habits^29–31^. The ability to activate, monitor, inhibit, maintain, and/or adapt one's behavior, attention, emotions, and cognitive strategies in response to internal cues, as well as external ones from feedback from others and environmental stimuli, plays an important role in self-regulation^27,32–34^. Of these capacities, Emotional Self-Regulation (ESR), defined as the ability to shape one's emotions, when one experiences them, and how they are expressed^35,36^ plays a key role in the acquisition and maintenance of healthy habits in adolescents, including the practice of physical activity^17,37,38^.

Previous research conducted among adolescents has demonstrated positive connections between PAH, ESR, and SWB. Specifically, emotional control techniques have been found to be linked to both PAH and SWB^39–42^. In a study by López-Gil et al.^17^, higher levels of physical exercise were positively correlated with increased ESR in adolescents, and the authors speculate that physical activity is associated with the development of resources that are relevant to autonomy and positive affect^40,41^.

A systematic review and meta-analysis conducted by Rodríguez et al*.*^43^ analyzed cross-sectional and experimental studies with an emphasis on the relationship between well-being and self-regulation. One of the investigations highlighted in this review was the study by Garcia et al*.*^42^, which involved a sample of adolescents and found a favorable association between SWB and ESR. Furthermore, a longitudinal study revealed a constant and meaningful link between ESR and SWB throughout adolescence. The authors also point out that individuals' perceptions of their SWB and coping mechanisms increase as their level of self-regulation does^44^. These strategies correspond to the cognitive and behavioral efforts required to manage dynamic demands that may be internal or external and are intended to lessen or completely eradicate negative feelings associated with stressful circumstances or conditions, such as social support, belonging, and avoidance^45^.

The ESR has been previously suggested as a potential mediator in the relationship between physical activity and overall well-being^46^. Despite its close relationship with both phenomena^47,48^, it has not been considered as a possible underlying mechanism that mediates the current association between PAH and SWB, particularly in adolescents. Therefore, it is of particular research interest to determine whether ESR is a mechanism by which physical activity is related to SWB^49^. This is especially relevant during adolescence when environmental issues related to the complexity of social experiences and interactions demand a higher level of cognitive processing and emotional regulation resources^30^. The ESR is particularly sensitive throughout adolescence since it has an association with mental health issues that emerge during this stage, such as depression, anxiety, and eating disorders, while the neurological system is still developing^50,51^.

Conversely, during this period, a slow and continuous decline in physical activity habits, subjective well-being, and other factors is observed^52–54^. Given that Chile has considerable economic and gender inequalities^55^, which, along with age, affect PAH^56,57^ and SWB^58^, exploring these phenomena in Chile may be particularly pertinent^59^. Previous studies that examined the relationship between SWB and PAH in Chile revealed substantial differences when controlling for gender, age, and socioeconomic status^60–62^.

In this sense, our study aims to make several contributions. Firstly, we aim to analyze the potential mediating effect of ESR on the relationship between PAH and SWB in adolescents, thus expanding our understanding of the underlying mechanisms involved in this relationship. Second, our research intends to further understanding of the association between physical exercise habits and subjective well-being in Chilean teenagers by taking into account social and demographic characteristics. Our findings could be used to develop initiatives or public policies that boost young people's well-being.

We hypothesize that ESR in adolescents will be positively associated with PAH and SWB and will act as a mediator in the relationship between PAH and SWB. Moreover, we expect that this mediation will be influenced by demographic factors such as gender, age, and socioeconomic status.

## Methods

### Participants

The sample consisted of 9585 participants, comprising 4922 females (51.4%) and 4663 males (48.6%), who were adolescents aged between 10 and 19 years (M = 13.88, SD = 2.08). At this point it should be mentioned that of the total of 10,152 adolescents surveyed, 567 (5.59%) were discarded, for not completely responding one or more scales of the study. The participants were students from the 5th year of primary school to the 4th year of secondary school, drawn from various types of educational establishments (public, subsidized, and private) located in all regions of Chile, spanning from the northern region of Arica to the southern region of Magallanes. The sampling was carried out using a probabilistic, two-stage, and stratified method. The first probabilistic sampling unit was the school, taking into account the different types of schools, and the second unit was the school year selected within each level. The Chilean Ministry of Education's official list of educational establishments for 2017 was used as the sampling frame.

### Data collection method

Schools that were randomly selected to participate in the study were contacted. The scales were administered as part of a broader instrument that included other scales throughout the 2017 academic year, during regular school hours. The measurements were conducted by a trained group under controlled circumstances. The students were asked to participate in the study while a teacher was present, and they filled out the surveys during a routine class with the assistance of the researchers.

### Instruments

#### Subjective well-being (SWB)

To assess subjective well-being among adolescents, we utilized the 7-item Personal Wellbeing Index School Version (PWI-SC)^63^. The psychometric properties of this scale have been previously examined in Chile, establishing its reliability and validity^64^. The scale comprises 11 levels, ranging from 0 (totally dissatisfied) to 10 (totally satisfied), and includes various life domains, such as standard of living, personal health, achievement in life, personal relationships, personal safety, feeling part of the community, and future security. The total score was computed as the average of the scores of the items that inquired about the participants' satisfaction with their possessions, health, competence, relationships, safety, activities outside their home, and future prospects. In this study, the scale's reliability was evaluated using Cronbach's alpha, which considered the seven items, and was found to be 0.86.

#### Physical activity habits (PAH)

The Eating and Physical Activity Habits Questionnaire for Schoolchildren, developed and validated by Guerrero et al*.*^65^, was utilized to assess Physical Activity Habits (PAH) and consisted of two dimensions and a total of 27 items, of which 18 items were intended to measure participants' eating and nutrition habits and 9 items were designed to assess their level of physical activity. Only five items, specifically assessing patterns of physical activity, were included in this study, while those measuring patterns of inactivity were excluded. Responses ranged from “Never or < once a month” (1 point) to “daily” (5 points). The total score calculated for our study was obtained from the mean of the five items that included the questions: “I do physical activities and/or sports with my family”, “I play in the park, garden, or playground with other children”, “At recess time, I engage in some kind of sport or physical activity”, “I practice some form of physical activity or sport in addition to the one I do in Physical Education and Sports”, and “I walk at least 15 min per day” (PAH derived from commuting). The meaning of physical activity and other terms was previously clarified in each class group. Cronbach's Alpha reliability score for this portion of the scale in Chilean adolescents was 0.71^66^, which is considered satisfactory^67^. The Cronbach's Alpha values for the entire instrument were 0.81 and 0.76 for the dimensions of eating and nutrition habits and physical activity habits, respectively.

#### Emotional self-regulation (ESR)

Consideration was given to the self-regulatory aspect of emotional regulation in this study. To measure emotional self-regulation, a scale based on Moilanen's self-regulatory inventory^34^ was used. This scale consists of five items specifically related to the control of emotions and short-term goals, such as activation, inhibition, monitoring, and perseverative adaptation. The items include: "I am usually able to do something that makes me feel better when I am sad", "I can refocus on what I was doing when I am interrupted", "I can calm down when I am excited or angry", "I can adapt and do something different to achieve my goal when something doesn't go as planned", and "I can maintain a calm demeanor when I am arguing with someone". Participants reported their responses on a 5-point Likert-type scale ranging from 1 (never) to 5 (very often). The total scale score was calculated by obtaining the mean score of all items. Higher scores indicate greater self-regulation. The internal consistency of this scale has been previously explored through Cronbach's Alpha in Chilean children and adolescents, resulting in a value of 0.82^17,68^, which is considered satisfactory.

#### Socioeconomic status (SES), gender and age

Our study used the School Vulnerability Index (SVI) to determine the socioeconomic status (SES) of the participants. The SVI is calculated for each educational facility based on factors such as family income, housing, the number of members, parents' educational attainment, and other social characteristics. It corresponds to a classification of low, medium, or high based on the proportion of students in the institution who meet the requirements of the National Board of School Aid and Scholarships to be classified as vulnerable. This measure is considered an indirect but reliable way of measuring SES^69,70^. In our study, students who attended an establishment with a high SVI (SVI = 1) were considered vulnerable, while students attending an establishment with medium or low SVI were considered non-vulnerable (SVI = 0). This is because the public education system in the country includes facilities with high SVI, which correspond to students who are more economically and socially vulnerable. The other types of vulnerable schools are those with partial or complete private system contributions. To establish gender, participants were given two options to respond with (0 = boys; 1 = girls). Age was measured by asking participants their age (in years) at the time of answering the questionnaire and was considered as both a continuous and categorical variable (early adolescence: 10–12 years of age; middle: 13–15 years of age; late: 16–20 years)^2^. Both gender and SVI were considered as dichotomous categorical variables. The distribution of the sample according to the sociodemographic factors considered is detailed in Table 1.

**Table 1:** Participants’ characteristics (*n* = 9585).

Characteristics	N (%)
Girls	4922 (51.4)
Boys	4663 (48.6)
Vulnerable	4634 (48.3)
Non-vulnerable	4951 (51.7)
10–12 years old	2866 (29.9)
13–15 years old	4237 (44.2)
16–19 years old	2482 (25.9)

### Statistical analysis

After establishing and cleaning the database, descriptive analyses were performed for the main study variables. Then, mean difference tests were performed for these variables, considering the control variables; gender (0 = man, 1 = woman), age (1 = early adolescence, 2 = middle adolescence, 3 = late adolescence) and SVI (0 = high, 1 = low), using the T Student and ANOVA tests with their respective post hoc analyses and Cohen’s d effect size (ES). The criteria to interpret the magnitude of the effect size was as follows: Cohen's (*d*) criteria: small (0.2), moderate (0.5) and large (0.8)^71^. Correlations between the study variables were also calculated using Pearson's correlation coefficient. Correlations between the study and sociodemographic variables were calculated using Spearman's correlation coefficient. The strength of the correlations was determined using Hopkins^72^ recommendations: trivial (< 0.1), small (0.1–0.3), moderate (0.3–0.5), high (0.5–0.7), very high (0.7–0.9) or practically perfect (> 0.9).

Subsequently, to contrast the study hypothesis, multivariate simple mediation models were developed^73,74^, in which students' subjective well-being (SWB) was considered as the dependent variable, physical activity habits (PA) as the independent variable, and self-regulation as the mediating variable. The individual control variables gender (categorical), age (continuous) and SVI (categorical) were additionally incorporated in the model. In the case of the categorical variables, dummy variables (dummies) were constructed so that they could be included in the respective models. Both independent and control variables were selected based on their theoretical and empirical relevance to this research. A bootstrap CI of BCa based on 5000 samples was used to calculate confidence intervals for all models used. All analyses were performed with IBM-SPSS version 25 software (IBM Inc., Armonk, NY. USA) and its PROCESS tool^73^.

### Ethical approval

The study adhered to ethical guidelines and was approved by the Bioethics Committee of the Pontificia Universidad Católica de Valparaíso, as evidenced by resolution BIOEPUCV-H 427-2021 issued in April 2017. Prior to the self-report application, an informed consent was obtained from each participant and their guardians, and these records were collected by the researchers. The confidentiality and privacy of each participant's data were ensured, and participation in the study was completely voluntary.

## Results

### Descriptive statistics

Table 2 provides descriptive statistics for all the variables taken into consideration for the study. Physical activity habits were shown to have the lowest mean scores in proportion to their theoretical maximum. Adolescents' low levels of physical activity habits were evident from this. The average ESR score was greater, and there was a marginally smaller dispersion. The variable that, when compared to the mean, showed the least amount of variation in adolescents is SWB, which was measured as having the greatest mean score in comparison to the theoretical maximum.

**Table 2:** Total scores for PAH, SWB and ESR.

Variable	Max	Min	Mean	SD
Physical activity habits	5	1	2.84	1.00
Subjective well-being	10	0	8.25	1.65
Emotional self-regulation	5	1	3.54	0.89

*SD* standard deviation.

### Comparative and correlational analysis

Table 3 shows the mean differences in PAH, SWB, and ESR for the control variables. Statistically significant differences were observed when comparing boys and girls with respect to their PAH and ESR. In all cases, the mean scores were higher in boys. When comparing the means of the study variables by age group, significant differences were found in PAH and the level of SWB. For both of these variables, the mean score obtained on the scales was higher in the early adolescence group (10–12 years old) and gradually decreased in the other two age groups. Only for ESR, the mean score of late adolescence (16–19 years old) was higher than in middle adolescence (13–15 years old).

**Table 3:** Mean differences and standard deviation in PAH, SWB and ESR for each control variable.

S. no	Boys	Girls	Test (p value)	*d*	10–12 years old	13–15 years old	16–19 years old	Test (p value)	*d*	Non vulnerable schools	Vulnerable schools	Test (p value)	*d*
PAH	3.07 ± 0.99	2.62 ± 0.96	t = − 22.51 (< 0.001)	0.46	3.22^b,c^ ± 1.03	2.74^a^ ± 0.96	2.72^a^ ± 0.97	f = 211.07 (< 0.001)	0.21	2.85 ± 0.98	2.82 ± 1.02	t = 4.58 (< 0.001)	0.10
SWB	8.3 ± 1.63	8.19 ± 1.66	t = − 3.53 (< 0.001)	0.07	8.64^b,c^ ± 1.38	8.19^a,^^c^ ± 1.64	8.09^a,b^ ± 1.74	f = 82.95 (< 0.001)	0.14	8.33 ± 1.58	8.17 ± 1.69	t = 1.27 (0.201)	0.03
ESR	3.65 ± 0.86	3.42 ± 0.91	t = − 12.41 (< 0.001)	0.26	3.61^b^ ± 0.90	3.44^a,c^ ± 0.90	3.57^b^ ± 0.88	f = 28.23 (< 0.001)	0.09	3.55 ± 0.88	3.52 ± 0.91	t = 1.27 (0.205)	0.03

Date are mean ± SD, a,b,c post hoc significant p < 0.001, *d,* Cohen’s effect size. *PAH* physical activity habits, *SWB* subjective well-being, *ESR* emotional self-regulation.

Finally, the comparative analysis of the study variables based on the SVI showed significant differences only for SWB. On the other hand, there were no differences in PAH or ESR of adolescents with respect to their level of school vulnerability.

Table 4 shows the bivariate correlations between the studied variables. In this regard, a small and positive correlation was observed between PAH, SWB, and PAH and SWB and ESR as perceived by adolescents. Likewise, a positive and moderate relationship was observed between subjective well-being (SWB) and perceived emotional regulation skills (ESR).

**Table 4:** Correlation matrix for the studied variables.

S. no	1. PAH	2. SWB	3. ESR
1. Physical activity habits	1		
2. Subjective well-being	0.270**	1	
3. Emotional self-regulation	0.239**	0.361**	1

All correlation corresponds to Pearson´s coefficients (r). **The correlation is significant at the 0.01 level (bilateral).

Table 5 shows that the correlations between the studied variables and the sociodemographic variables displayed different results. In the case of SWB, there was a positive and significant correlation between SWB and gender (0 = girls, 1 = boys; rho = 0.04). On the other hand, the relationship was negative with age (rho = − 0.16) and SVI (rho = − 0.04); as age and SVI increase, SWB is lower. The relationship between PAH and gender was positive (rho = 0.23). In contrast, a negative relationship was observed between PAH and age, as age increased, PAHs decreased (rho = − 0.29). No relationship was observed between PAHs and SVI in the sample of adolescents examined. Finally, ESR showed a positive relationship with gender (rho = 0.13). A negative relationship was found between ESR and age (rho = − 0.03), as age increase ESR is lower. No significant relationship was observed between ESR and SVI. Despite the above, it is necessary to point out that all significant correlations found in these analyses were of trivial to small magnitude.

**Table 5:** Correlation matrix for SWB, PAH and ESR and the sociodemographic variables.

Correlation	Gender	Age	SVI
Physical activity habits	0.23**	− 0.29**	− 0.02
Subjective well-being	0.04**	− 0.16**	− 0.04**
Emotional self-regulation	0.13**	− 0.03*	− 0.01

All correlation corresponds to Spearman’s coefficients (rho). *SVI* school vulnerability index (0 = no vulnerable, 1 = vulnerable). **Correlation is significant at the 0.01 level (two-tailed). *Correlation is significant at the 0.05 level (two-tailed).

### Mediation analysis

**Figure 1:**
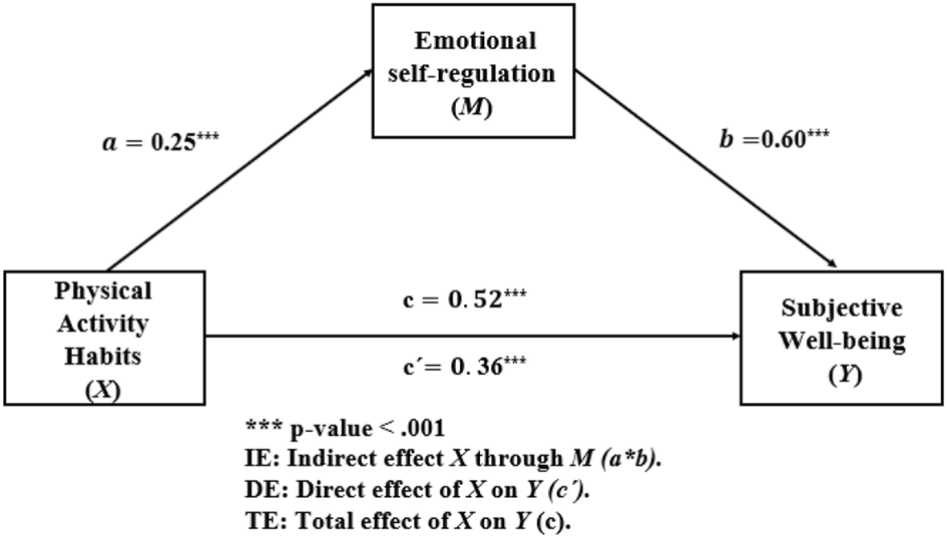
Mediation model of emotional self-regulation between physical activity habits and subjective well-being.

This section presents the results of a simple mediation analysis. Mediation analysis is used to understand how the relationship between two variables can be divided into direct and indirect effects. It allows us to determine how much of the effect of the independent variable on the dependent variable is due to the effect of the independent variable on a mediating variable, and to the effect of this mediating variable on the dependent variable. As Hayes^73^ states, "a mediation model is used to introduce mechanics of path analysis and to demonstrate how a variable's effect on an outcome can be partitioned into direct and indirect effects that can be quantified using OLS regression" (p.77). In our study, we considered ESR as a potential mediator between PAH and SWB. Table 5 displays the results of the linear regression analysis of the two sub-models that make up the mediational study. One sub-model considers ESR as the dependent variable, and the other sub-model considers SWB as the dependent variable. The magnitude and direction of the effects are shown in Fig. 1.

As can be seen in the model in Fig. 1, the total effect of PAH on SWB was statistically significant [TE: b = 0.52, 95% BCa CI (0.47, 0.56)]. Likewise, when decomposing the total effect, it can be observed that the direct effect was also statistically significant [SD: b = 0.36, 95% BCa CI (0.32, 0.41)] and that the same occurs in the case of the indirect effect [IE: b = 0.15, 95% BCa CI (0.13, 0.17)]. Taking into account the aforementioned, it is clear that the model included partial mediation, wherein some of the effect of PAH on SWB followed a direct pathway and other portions followed an indirect pathway via ESR.

Only age showed a statistically significant association among the control variables in both sub-models, as shown in Table 6. The linear regression model that used ESR as the dependent variable had statistically significant results for gender, whereas the model that used SWB as the dependent variable had statistically significant results for SVI.

**Table 6:** Linear regression analysis for mediational model.

Consequent								
Antecedent	*M* (emotional self-regulation)				*Y* (subjective well-being)			
		Coeff	*SE*	*p*		Coeff	*SE*	*p*
*X* (physical activity habits)	*a*	0.25	0.01	< 0.001	*c'*	0.36	0.02	< 0.001
*M* (emotional self-regulation)		–	–	–	*b*	0.60	0.02	< 0.001
Gender		0.19	0.02	< 0.001		− 0.06	0.03	0.07
Age		0.01	0.01	0.02		− 0.08	0.01	< 0.001
SVI		-0.04	0.02	0.08		− 0.04	0.03	0.20
Constant	***i***_***M***_	2.54	0.09	< 0.001	**i**_**γ**_	6.20	0.15	< 0.001
		***R***^***2***^ = 0.20				***R***^***2***^ = 0.20		
		*F* (4, 9580) = 138.74, *p* < 0.001				*F* (5, 95,876) = 354.04, *p* < 0.001		

*SE* size effect, *SVI* school vulnerability index, *R*^*2*^ R-squared of model.

## Discussion/conclusions

This study aimed to contribute to the existing research on the relationship between PAH and SWB in adolescents by exploring the potential mediating role of ESR. Previous research has found a positive correlation between physical exercise and SWB in adolescents, which supports the current study's hypothesis. For instance, Bueker et al*.*^75^ conducted a systematic review and meta-analysis that revealed a positive association between physical exercise and SWB in observational and experimental research. The authors noted that the effects on SWB are more significant in experimental trials that involve systematic and directed physical exercise. Additionally, young adults who engaged in more than 150 min per week of moderate to strenuous physical activity demonstrated higher levels of life satisfaction. Similarly, Gómez-Baya et al*.*^76^ investigated the association between sports participation, physical activity, and life satisfaction among adolescents and found that this relationship was more prominent among female teenagers aged 13–16. Thus, this study aimed to add to this body of literature by examining the potential mediating role of ESR in the association between PAH and SWB among adolescents.

A positive correlation between PAH and ESR was observed among the adolescents examined in our study. Similar results were reported by López-Gil et al*.*^17^, who investigated the relationship between PHA and ESR in Chilean youth. The researchers found that physically active children and adolescents had significantly higher self-regulation means compared to their sedentary peers. They also suggest that improvements in physical activity may be associated with enhanced personal resources, such as self-acceptance, social connectivity, autonomy, positive affect, and life satisfaction, all of which are related to self-regulation. Costigan et al*.*^77^ concur that vigorous physical activity enhances positive affect and is inversely associated with negative affect. They also mention that emotional self-regulation may be one of the processes underlying the impact of routine physical activity on SWB. Hagger^22^ highlights the fact that routine physical activity patterns develop from repeated encounters in predictable environments and may play a critical role in regulating behavior. The results of our study also demonstrate a connection between the SWB and the SRE of adolescents. These findings are consistent with cross-sectional studies^42^ and meta-analyses^43^ that emphasize the substantial and favorable association between SWB and emotional self-regulation, particularly with emotional regulation strategies associated with acting and adhering to a process until objectives are met. Longitudinal studies provide further evidence of this association, highlighting coping mechanisms that increase the level of self-regulation^44,45^.

The aim of our study was to investigate the potential mediating effect of SRE on the relationship between PAH and SWB in adolescents. Our findings demonstrate a partial mediation effect, which highlights the importance of SRE in partially explaining the association between PAH and SWB in this population. To the best of our knowledge, there are currently no mediation studies available that examine the same variables of interest within the adolescent population. However, Garcia et al*.*^42^ conducted a study that closely resembles ours, but only involved a correlation analysis between PAH, SWB, and ESR. The correlation coefficients reported by these authors between PAH and ESR (r = 0.20, p < 0.05) and PAH and SWB (r = 0.24, p < 0.01) were statistically significant, despite using different scales for the measurements. This emphasizes the similarities in our findings with theirs, even in terms of the magnitude of the correlation coefficients.

The mediation effect found in our study was ultimately due to the positive and statistically significant association between PAH and ESR, which highlights the significance of SRE as a powerful resource for the formation of healthy lifestyle habits. In line with our findings, Melguizo-Ibáñez et al.^78^ reported that specifically, children and adolescents who engaged in more than three hours of weekly physical activity exhibited higher scores in emotional intelligence, including emotional regulation skills. López-Gil et al*.*^17^ also identified a significant relationship between PHA and ESR (r = 0.265, p < 0.001) in their study of Chilean youth, further reinforcing the close relationship between PAH and ESR. They suggest that increases in physical exercise could help develop personal resources such as autonomy, positive affect, and life satisfaction, which seem to be the mechanisms by which many physical activity-based therapies report having a positive impact on self-regulation. However, it is crucial to balance the growth of PAH and ESR precisely, as excessive and dangerous physical activity has been linked to a lack of or failure in the regulation of emotions. Goodwin et al*.*^79^ conducted a longitudinal study that revealed a link between such actions and obsessive–compulsive disorders and eating disorders, including anorexia.

In our study, age was the only control variable that demonstrated a statistically significant association in both sub-models. This finding is consistent with prior research on subjective well-being (SWB) in adolescents, as reported by González-Carrasco et al*.*^58^, who found age-related differences in various SWB measures. In a longer follow-up study on SWB development in teenagers, the authors confirmed a declining trend in SWB with age, particularly among girls^8^. National surveys on SWB in teenagers between ages 12 and 18 have reported substantial variations in SWB based on age, gender, and socioeconomic status. Boys had higher mean levels of well-being than girls, and those who were older and less vulnerable at school reported higher levels of perceived well-being^60^. The general trend in the literature up to this point establishes that subjective well-being decreases with age in adolescents; however, some previous studies conducted in Chile warn of discrepancies with this trend. In this regard, a validation study conducted with Chilean adolescents refers to the fact that around 70% of them report consistently high levels of well-being^64,80^. Similarly, earlier studies in Chile, such as Oyanedel et al*.*^69^, caution that especially during the transition from childhood to early adolescence, differences in subjective well-being may not be observed. The author attributes these findings to the so-called "optimism bias", which posits that when asked about personal satisfaction, young individuals tend to report significantly higher levels of satisfaction than dissatisfaction. Previously, Casas et al*.*^81^ described this phenomenon, primarily associating it with child and adolescent populations.

On the other hand, previous national^61^ and international reports on physical activity habits (PAH) have shown that activity levels gradually and consistently decline during adolescence^62^. It is noteworthy that socioeconomic vulnerability, as well as age and gender, are significant predictors of PAH in adolescents, given that unfavorable environments for physical activity may be associated with lower levels of activity. Although self-regulation was also associated with age (specifically, stage of adolescence), we did not observe a declining trend in self-regulation as was the case for PAH and SWB. The mechanisms of decision-making and long-term planning are influenced by brain systems that process emotions and cognitive control, which are not equally developed during early and middle adolescence^30,82^. Age and gender differences in emotional regulation appear to arise from a combination of biological differences, socialization, and the influence of social context, timing, and cultural expectations^83,84^.

The study results suggest that SRE partially mediates the relationship between PAH and SWB in adolescents. This highlights the importance of promoting physically active lifestyles to establish and maintain healthy habits in this population. Engaging in physical activity can also facilitate emotional regulation in adolescents, promoting the use of both short- and long-term strategies. To improve teenage subjective well-being, public programs and policies should strive to foster the development of PAH and SRE (SWB). In this regard, programs such as *"Physically Active Learning"* (PAL) in Europe^85^ and *"Comprehensive School Physical Activity Program"* (CSPAP) in the United States^86^ serve as examples of physical activity promotion programs. On one hand, these programs emphasize the role of physical activity as a strategy in generating learning processes, and on the other, they foster the development of a school culture that promotes physical activity for overall well-being throughout life. Both initiatives promote physical activity habits during adolescence and recognize the value of this stage in establishing healthy habits for adulthood.

However, the study has some limitations. Firstly, it did not consider the subjects' level of maturity. Secondly, the socioeconomic status was measured using a vulnerability index that may not reflect the individual reality of each student. Lastly, the study had a cross-sectional design, making it unable to provide evidence on the predictive role of PAH on SWB, and the proposed causality between these variables is based on theoretical assumptions.

Future studies could expand the current research by exploring additional variables that could serve as valid mechanisms to explain the relationship between subjective well-being and physical activity, such as self-efficacy or self-image, which have also been recognized in the literature as important factors associated with subjective well-being and physical activity habits in adolescents^47,87,88^. Furthermore, it would be beneficial to consider new relevant sociodemographic factors for the population in Chile, such as migration^87,89^, that could shed light on the underlying mechanisms linking physical activity and subjective well-being in adolescents, thus providing a more comprehensive understanding of this relationship.

## Data Availability

The datasets used and analysed during the current study available from the corresponding author on reasonable request.
